# Clinical predictors of type II endoleaks after EVAR: a systematic review of risk and protective factors

**DOI:** 10.1093/bmb/ldag035

**Published:** 2026-10-19

**Authors:** Giulia Colonna, Chiara Fasulo, Mario Antonelli, Anna Santini, Costanza Morucci, Ginevra Mazzetti, Luigi Rizzo, Maurizio Taurino, Pasqualino Sirignano

**Affiliations:** Vascular and Endovascular Surgery Unit, Sant'Andrea Hospital of Rome, Department of Molecular and Clinical Medicine, “Sapienza” University of Rome, Via di Grottarossa, 1035, 00189 Roma RM, Rome, Italy; Vascular and Endovascular Surgery Unit, Sant'Andrea Hospital of Rome, Department of Molecular and Clinical Medicine, “Sapienza” University of Rome, Via di Grottarossa, 1035, 00189 Roma RM, Rome, Italy; Vascular and Endovascular Surgery Unit, Sant'Andrea Hospital of Rome, Department of Molecular and Clinical Medicine, “Sapienza” University of Rome, Via di Grottarossa, 1035, 00189 Roma RM, Rome, Italy; Vascular and Endovascular Surgery Unit, Sant'Andrea Hospital of Rome, Department of Molecular and Clinical Medicine, “Sapienza” University of Rome, Via di Grottarossa, 1035, 00189 Roma RM, Rome, Italy; Vascular and Endovascular Surgery Unit, Sant'Andrea Hospital of Rome, Department of Molecular and Clinical Medicine, “Sapienza” University of Rome, Via di Grottarossa, 1035, 00189 Roma RM, Rome, Italy; Vascular and Endovascular Surgery Unit, Sant'Andrea Hospital of Rome, Department of Molecular and Clinical Medicine, “Sapienza” University of Rome, Via di Grottarossa, 1035, 00189 Roma RM, Rome, Italy; Vascular and Endovascular Surgery Unit, Sant'Andrea Hospital of Rome, Department of Molecular and Clinical Medicine, “Sapienza” University of Rome, Via di Grottarossa, 1035, 00189 Roma RM, Rome, Italy; Vascular and Endovascular Surgery Unit, Sant'Andrea Hospital of Rome, Department of Molecular and Clinical Medicine, “Sapienza” University of Rome, Via di Grottarossa, 1035, 00189 Roma RM, Rome, Italy; Vascular and Endovascular Surgery Unit, Sant’Andrea Hospital of Rome, Department of General and Specialistic Surgery, “Sapienza” University of Rome, Piazzale Aldo Moro, 5, 00185 Roma RM, Rome, Italy

**Keywords:** aortic aneurysm, EVAR, endoleaks, risk factors

## Abstract

**Introduction:**

The present systematic review seeks to examine clinical factors potentially associated with endovascular aortic aneurysm repair (EVAR) failure due to type II endoleaks (TIIEL).

**Source of data:**

Relevant literature was sourced from PubMed, including all studies regarding clinical features possibly linked to TIIEL, written in English. Studies focused on anatomical predictors, case reports, and abstracts were excluded. The literature search used keywords related to clinical characteristics, comorbidities, and demographics common in EVAR patients.

**Areas of agreement:**

EVAR is the preferred treatment for abdominal aortic aneurysms in eligible patients. The most common complication is endoleak (persistent filling of the sac). TIIEL is caused by retrograde blood flow from aortic side branches and is the most frequent complication, occurring in 16%–50% of cases.

**Areas of controversy:**

Many studies have explored anatomical predictors of TIIEL, but limited research is available on clinical factors that may contribute to its occurrence.

**Growing points:**

Protective factors against TIIEL included statin therapy, salicylate therapy, smoking, ankle/brachial systolic blood pressure index <0.87, and chronic obstructive pulmonary disease. Conversely, anticoagulation therapy, inflammatory status, and advanced age were associated with an increased risk of TIIEL.

**Areas timely for developing research:**

Further prospective research is needed to validate the presented association between clinical factors and EVAR failure due to TIIEL, to allow risk stratification and tailored follow-up strategies for selected groups of patients.

## Introduction

Endovascular aneurysm repair (EVAR) is currently the preferred treatment for abdominal aortic aneurysm (AAA) in suitable patients, with better short-term outcomes compared to open surgery.^1–3^ Nevertheless, *EVAR* treatment is characterized by a high rate of complications and reinterventions. The complications associated with EVAR include rupture, occlusion, graft migration, kinking, infolding, infection, and, most frequently, endoleaks.^4^ Endoleak refers to persistence of the blood flow outside the lumen of the endograft, resulting in continued perfusion of the aneurysm sac.^5^ Regarding the origin of the endoleak, endoleaks are classified in five types. More details are explained in Fig. 1. Type II Endoleaks (TIIEL) are due to persistence of blood flow from collateral arteries into the aneurysm sac.^5–7^ Based on the time of manifestation, endoleaks can be divided in primary endoleaks, within 30 days from intervention and secondary endoleaks, after 30 days. TIIEL are the most frequently observed with a rate of occurrence in 10.2% of patients after EVAR, often associated with continued retrograde perfusion of the aneurysm sac by the inferior mesenteric artery (IMA) and lumbar arteries.^8^^,^^9^ In contrast to type I and type III endoleaks, for which intervention is always warranted, the management of TIIEL remains controversial, since more than a half can solve spontaneously.^10^ European and Italian Society of Vascular and Endovascular Surgery Guidelines suggest treating this type of endoleak only in the presence of significant aneurysm sac growth (>10 mm), provided alternative causes including type 1 and type 3 endoleaks have been excluded.^11^^,^^12^ Nevertheless, TIIEL can lead to aneurysm rupture in ~1% of cases.^10^ Given the high incidence and variable outcomes of TIIEL, identifying patients at increased risk of developing this complication after EVAR has become increasingly important. Several studies have explored anatomical factors that may predispose to TIIEL, such as the number and location of vessels potentially maintaining perfusion of the aneurysm sac.^10^^,^^13^^,^^14^ However, evidence regarding clinical characteristics associated with a higher risk of TIIEL development remains limited.

**Figure 1:**
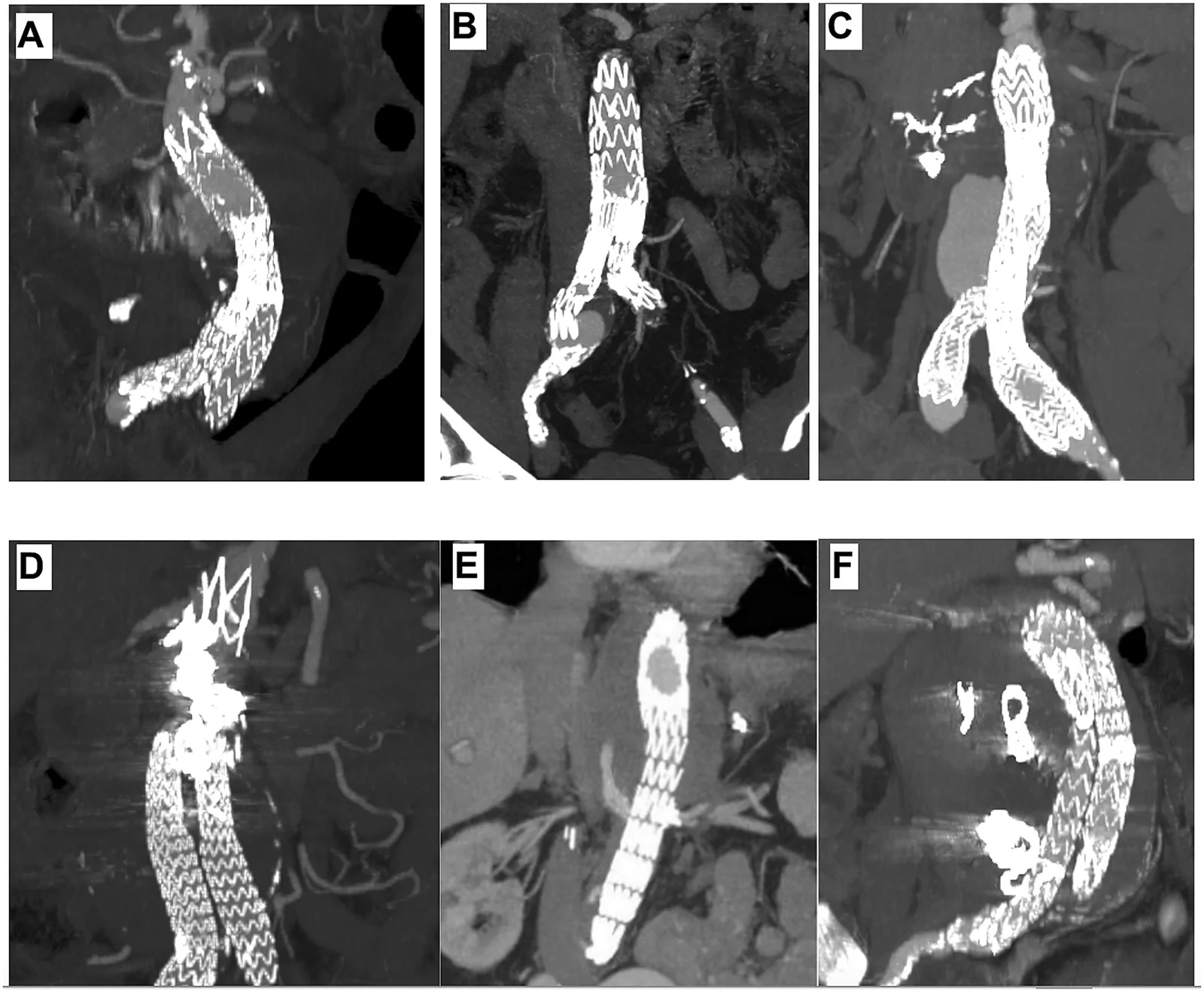
Endoleaks classification based on their origin. (A) Type IA: Inability to form a full circumferential seal at the proximal end of the endograft; (B) type IB: Inability to form a full circumferential seal at the distal end of the endograft; (C) type II: Persistence of blood flow from collateral arteries into the aneurysm sac; (D) type III: Structural compromission of the implanted graft such as junctional separation of components, migration, holes and tears in the endograft; (E) type IV: Porosity of the graft fabric (F) type V: Endotension inside the graft due to continued sac enlargement without a confirmed cause.

The primary aim of this review is to systematically collect and synthesize the existing literature on clinical factors that may be linked to EVAR failure resulting from TIIEL.

## Methods

### Data search study selection

A systematic search was performed on 6 December 2024. The electronic bibliographic database PubMed was searched using both Medical Subject Headings (MESH) terms and free text terms between years 2005 and 2024. In the search strategy, the terms “EVAR” and “Type II Endoleak” were combined, and corresponding search terms were also included. Inclusion criteria included: (i) patients over 18 years of age; (ii) AAA treated in urgent/elective regime; (iii) TIIEL occurrence during follow-up (iv) studies investigating possible clinical features linked with TIIEL (v) articles written in English. The types of publications studied were limited to reviews, meta-analysis, retrospective studies, prospective studies and observational studies. Exclusion criteria included (i) articles regarding anatomical features possibly related to TIIEL (ii) case report (iii) studies observing other types of endoleaks.

The search string was (("EVAR"[All Fields]) AND "endoleak"[MeSH Terms] OR "endoleak"[All Fields] OR "endoleaks"[All Fields]) AND ("endoleak"[MeSH Terms] OR "endoleak"[All Fields] OR "endoleaks"[All Fields]) AND ("hypertense"[All Fields] OR "hypertension"[MeSH Terms] OR "hypertension"[All Fields] OR "hypertension s"[All Fields] OR "hypertensions"[All Fields] OR "hypertensive"[All Fields] OR "hypertensive s"[All Fields] OR "hypertensives"[All Fields]) AND ("sex"[MeSH Terms] OR "sex"[All Fields]) AND (‘pulmonary disease, chronic obstructive’[MeSH Terms] OR ("pulmonary"[All Fields] AND "disease"[All Fields] AND "chronic"[All Fields] AND "obstructive"[All Fields]) OR ‘chronic obstructive pulmonary disease’[All Fields] OR "copd"[All Fields]) AND ("diabete"[All Fields] OR "diabetes mellitus"[MeSH Terms] OR ("diabetes"[All Fields] AND "mellitus"[All Fields]) OR "diabetes mellitus"[All Fields] OR "diabetes"[All Fields] OR "diabetes insipidus"[MeSH Terms] OR ("diabetes"[All Fields] AND "insipidus"[All Fields]) OR "diabetes insipidus"[All Fields] OR "diabetic"[All Fields] OR "diabetics"[All Fields] OR "diabets"[All Fields]) AND ("inflammation"[MeSH Terms] OR "inflammation"[All Fields] OR "inflammations"[All Fields] OR "inflammation s"[All Fields]) AND ("NLR"[All Fields]) AN D ("PLR "[All Fields]) AND ("MMP "[All Fields]) AND ("anticoagulants"[Pharmacological Action] OR "anticoagulants"[MeSH Terms] OR "anticoagulants"[All Fields] OR "anticoagulant"[All Fields] OR "anticoagulate"[All Fields] OR "anticoagulated"[All Fields] OR "anticoagulating"[All Fields] OR "anticoagulation"[All Fields] OR "anticoagulations"[All Fields] OR "anticoagulative"[All Fields])).

Additional studies were identified through the bibliographies of the articles retrieved using the present research string.

Two independent reviewers (G.C., C.F.) screened the records for inclusion. Articles that met the eligibility criteria were retrieved in full text and reassessed. Studies for which full-text versions were unavailable were excluded. Discrepancies in eligibility were resolved through discussion and, when necessary, by a third review author (P.S.).

### Data synthesis and statistical analysis

Data were analyzed using the Statistical Package for the Social Sciences version (SPSS), version 27.0 (IBM Corp, Armonk, NY, USA, 2020). Demographic and clinical data were displayed as mean (±SD) for normally distributed data, as median (25th–75th percentile, interquartile range) for non-parametric data, and as frequencies (percentages) for the categorical data. Due to the small sample size and lack of uniform follow-up data, no statistical comparison between treatment modalities was performed.

### Study objective

The primary objective of this systematic review is to summarize the clinical factors that have been investigated as potential determinants of TIIEL development after EVAR, and to classify them, according to their reported association with TIIEL, as potential risk or protective factors. Specifically, this review focuses on patient-related characteristics, including demographic variables, comorbidities, lifestyle habits, inflammatory markers, laboratory markers, genetic polymorphisms, and perioperative medical therapies. The secondary objective is to identify subgroups of patients at increased risk of TIIEL development who may benefit from tailored postoperative surveillance.

## Results

### Study characteristics

From the 74 studies initially identified by the present search strategy, 17, published between 2002 and 2024, were included in the present review. The PRISMA flow diagram summarizing the studies selection process are shown in Fig. 2. Details regarding the selected studies are summarized in Table 1.

**Figure 2:**
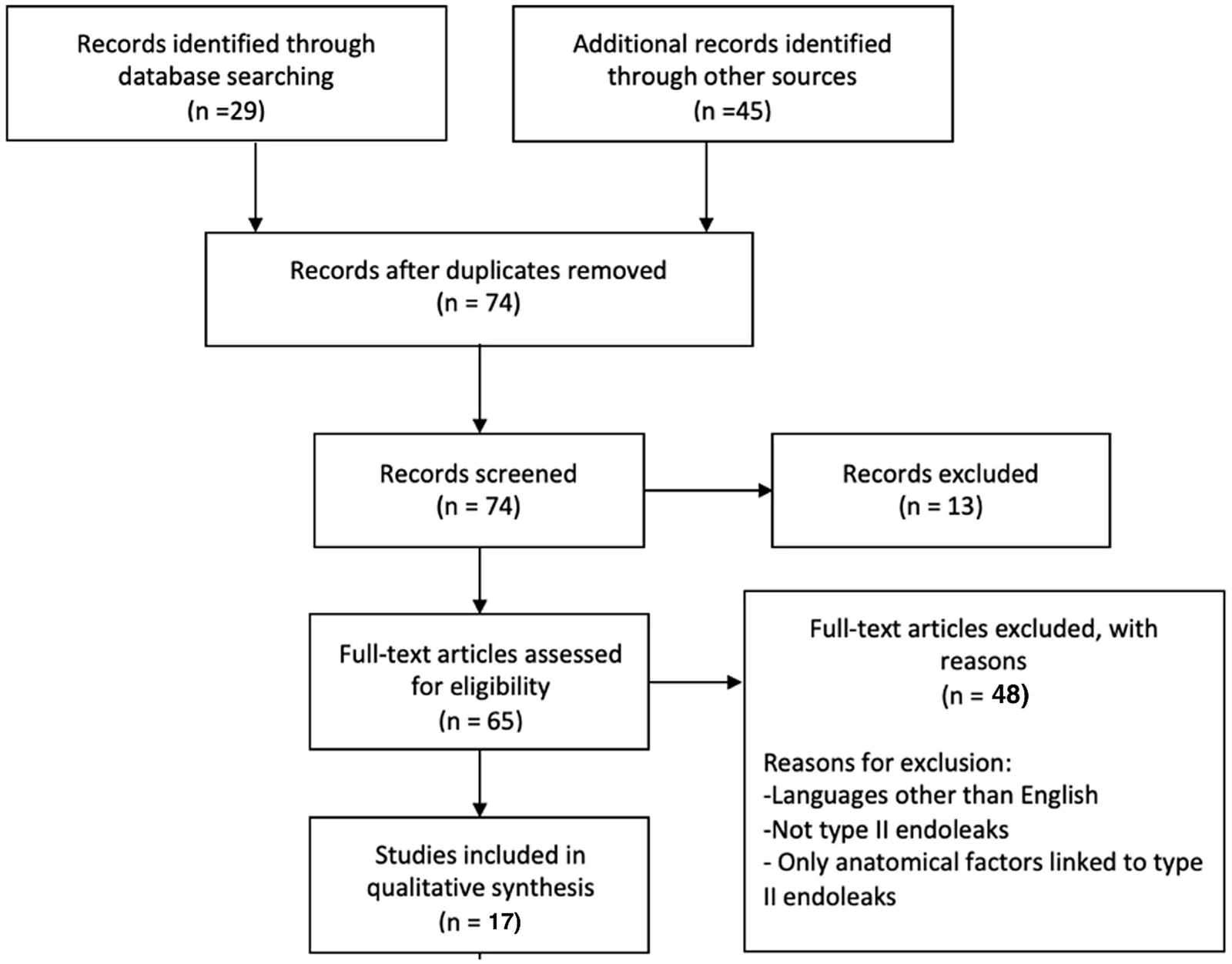
PRISMA flow diagram showing screening and selection of studies for the present review.

**Table 1:** Study characteristics.

First author	Publication year	Study design
Van Marrewijk, C. J.^15^	2004	Retrospective study
Bobadilla J.^16^	2010	Narrative review
Koole D.^17^	2012	Narrative review
Lazarides M.^18^	2014	Meta-analysis
De Rango P.^19^	2014	Narrative review
Pini R.^20^	2015	Retrospective study
Fujimura N.^21^	2016	Retrospective study
Alvarez Marcos F.^22^	2017	Retrospective study
Guo Q.^23^	2017	Meta-analysis
Seike Y.^24^	2017	Retrospective study
Courtois A.^25^	2019	Prospective study
Liu Z.^26^	2019	Retrospective study
Flohr T.^27^	2021	Retrospective study
Kong D.^28^	2022	Retrospective study
D’Oria M.^29^	2022	Retrospective study
Kontopodis N.^30^	2023	Systematic review
Bruijn L.^31^	2024	Systematic review

### Clinical risk factors, potential factors and unrelated factors linked to TIIEL

Eighteen clinical factors were identified as possibly related to TIIEL occurrence, and they were divided by association with TIIEL in “Protective Factors” and “Risk Factors”.

Clinical factors associated with an increased probability of TIIEL after EVAR are shown in Table 2.

**Table 2:** Risk factors for TIIEL.

Factor	Author	Main finding	*P* value
Anticoagulant therapy	Kontopodis^30^	Anticoagulation was associated with a higher risk of persistent TIIEL and impaired aneurysm sac regression after EVAR	<.001
	Lazarides^18^	Long-term anticoagulation was associated with a higher incidence and persistence of TIIEL after EVAR	
	Kong^28^	Anticoagulation was associated with an increased incidence of TIIEL and reduced likelihood of aneurysm sac regression.	.023
	De Rango^19^	Chronic anticoagulation was associated with a higher incidence of endoleaks during follow-up after EVAR.	.02
	Bobadilla^16^	Warfarin therapy was associated with a significantly higher incidence of endoleaks and increased aneurysm sac volume.	.02
	Flohr^27^	Oral anticoagulation was associated with an increased risk of persistent or late endoleaks after EVAR.	<.001
	Bruijn^31^	Anticoagulant therapy was associated with increased TIIEL persistence and unfavorable sac remodeling.	.02
Warfarin Therapy	Seike^24^	Warfarin therapy was an independent predictor of persistent TIIEL and aneurysm sac enlargement.	.013
Anticoagulant + antiplatelet therapy	Kong^28^	Combined anticoagulant and antiplatelet therapy was associated with impaired aneurysm sac regression and increased TIIEL occurrence	.001
TIMP1 (nonTcarrier)	D’Oria^29^	The TIMP1 non-T carrier genotype was associated with adverse aneurysm sac remodeling and increased TIIEL risk	.05
Age > 80	Liu Z.^26^	Increasing age was independently associated with a higher risk of TIIEL-related reintervention	<.004
Age > 73	Van Marrewijk^15^	Older age was associated with a higher incidence of TIIEL after EVAR.	.002
	Guo^23^	Meta-analysis identified older age as a significant risk factor for TIIEL development.	<.001
Inflammation (FDG uptake)	Courtois^25^	Meta-analysis identified older age as a significant risk factor for TIIEL development.	.04

On the other hand, clinical factors such as statin therapy, antiplatelet therapy, peripheral artery disease (PAD) resulting in an Ankle-Brachial Index (ABI) <0.87, smoking habit, and COPD were described as potentially protective of TIIEL development after EVAR. More information is presented in Table 3.

**Table 3:** Protective factors for TIIEL.

Factor	Author	Main finding	*P* value
Statin therapy	Pini^20^	Statin therapy was associated with greater aneurysm sac shrinkage and diameter reduction during follow-up	0.16 (6 months) .001(36 months)
Antiplatelet Therapy (salicylates)	Alvarez^22^	Salicylate therapy was associated with a lower risk of aneurysm sac growth in patients with TIIEL compared with other antiplatelet regimens.	.007
Van Merrewijk	Van Marrewijk^15^	Antiplatelet therapy was associated with a lower occurrence of TIIEL after EVAR.	<.017
ABI < 0.87	Van Marrewijk	A lower ABI was associated with a reduced occurrence of TIIEL, potentially reflecting greater atherosclerotic involvement of collateral vessels.	.0002
Smoker status	Fujimura^21^	Active smoking was inversely associated with TIIEL occurrence, while non-smoking status was identified as an independent risk factor for TIIEL.	<.001
	Koole^17^	Active smokers showed a significantly lower incidence of TIIEL compared with former and non-smokers.	<.001

### Risk factors

#### Anticoagulation

As overall survival improves and EVAR procedures become more common, the prevalence of patients on long-term oral anticoagulation therapy is likely to increase. While antiplatelet therapy is routinely recommended following EVAR, anticoagulant therapy is frequently required in these patients due to coexisting cardiovascular conditions, particularly atrial fibrillation. However, it has been hypothesized that full anticoagulation, particularly with an International Normalized Ratio (INR) >2, may impair the thrombus formation of the excluded aneurysm sac and feeding side branches (lumbar arteries, IMA), thereby allowing persistent sac perfusion and enlargement after EVAR and contributing to the development and persistence of TIIEL.^15^^,^^16^ As shown in Table 2, eight of the included studies of the present review investigated the impact of anticoagulant therapy in TIIEL and concluded that anticoagulant therapy, both with warfarin and direct oral anticoagulants (DOAC), negatively impact sac remodeling and significantly increases the likelihood of such complication. The prospective study conducted in 2010 by Bobadilla *et al.* concluded that, after a mean follow-up of 2.1 years, the warfarin group of patients had a significant higher incidence of TIIEL and overall aneurysm sac volume increase,^17^ findings that align with the approximately two-fold increased incidence reported in other non-warfarin comparator studies.^15^^,^^18^^,^^19^ More recent evidence from Kong *et al.* and Flohr *et al.* indicates that similar adverse trends are also observed in patients treated with modern DOACs.^20^^,^^21^ The systematic reviews by Kontopodis *et al.* and Lazarides *et al.* confirmed this association across heterogeneous patient populations, where anticoagulation consistently remained a statistically significant predictor of persistent TIIEL across diverse study designs.^15^^,^^16^ However, it is worth to consider that in many of the included study, despite being consistently associated with an increased risk of TIIEL, warfarin and DOAC therapy were not associated with significantly higher reintervention rates. Therefore, it can be speculated that anticoagulation primarily affects sac physiology rather than inevitably leading to secondary interventions. In conclusion, anticoagulant therapy should be avoided after EVAR whenever possible and replaced with antiplatelet therapy. When anticoagulation cannot be discontinued due to underlying comorbidities (e.g. atrial fibrillation, mechanical valves), it should be considered as part of the prognostic stratification of patients subjected to EVAR, and this selected group of patients should undergo a close follow-up to prevent and promptly treat “malignant” TIIEL.

#### Age

Various studies included in the present review have investigated older age as a risk factor for TIIEL development.^22–24^ The physiological explanation for this association could be that age is associated with higher AAA diameter and disease history, favoring high-pressure aneurysm sac. Additionally, older patients frequently have more advanced atherosclerotic disease, possibly affecting collateral flow patterns.

The meta-analysis conducted by Guo *et al.* identified age as a significant predictor of TIIEL development (*P* < .001), together with anatomical features such as patent IMA, greater number of patent lumbar arteries and larger aneurysm diameter.^22^ Recently, Liu *et al.* demonstrated that age independently increases the likelihood of re-intervention in patients with established T2EL, with each additional year conferring a higher probability (17%) of adverse sac evolution requiring treatment.^23^ However, it is important to note that age may not act as an independent predictor of TIIEL but rather as a condition associated with other factors that primarily drive the increased risk of TIIEL. Taken together, these findings suggest that although age is not a consistent predictor of TIIEL onset, it likely contributes to vascular conditions that favor persistent sac perfusion and may affect the subsequent evolution or clinical relevance of T2EL, particularly the likelihood of requiring secondary intervention. It may be reasonable to consider elderly patients at higher risk for this complication and, consequently, to implement stricter surveillance after EVAR.

#### Inflammation state and metalloproteinases

Recent reports point out that TIIEL may be influenced not only by anatomical factors but also by an underlying inflammatory state of the aortic wall and by altered extracellular matrix remodeling. Courtois *et al.* demonstrated that patients with increased pre-operative aneurysm wall inflammation, analyzed through elevated fluorodeoxyglucose (FDG) uptake on pre-operative positron emission tomography/computed tomography (PET-CT), were significantly at higher risk of developing TIIEL after EVAR, supporting the hypothesis that inflammation may impair aneurysm sac thrombosis and promote persistent collateral perfusion.^25^ However, this finding originates from a single study specifically designed to evaluate local sac inflammation, and therefore FDG-PET/CT cannot be considered sufficient evidence of a broader or systemic inflammatory state, which remains yet to be demonstrated.

In addition, D’Oria *et al.* identified the mutation rs4898 of TIMP-1 non-T carriers was associated with TIIEL, probably due to alterations of matrix metalloproteinases and their inhibitors, which may affect the aneurysm sac healing process.^26^ These findings suggest that both inflammatory activity and genetically mediated differences in matrix remodeling may create a biological environment that favors the persistence of collateral flow and the development of TIIEL after EVAR. Nevertheless, data regarding a possible association between TIIEL occurrence and systemic inflammatory markers (e.g. C-reactive protein, procalcitonin), easier to measure compared to PET/CT and genetic profiling, are still lacking. For this reason, although genetic profiling and FDG-PET/CT may hold potential for pre-operative risk stratification, their current role remains limited, and further studies are needed to investigate the role of systemic inflammation in TIIEL before they can reliably identify patients who may require tailored postoperative surveillance.

### Protective factors

#### Statin therapy

In 2015, a retrospective study was conducted by Pini *et al.* on a poll of 756 patients to investigate a potential relation between TIIEL evolution and statin therapy, underlining its role in stabilizing the aneurysmatic sac. According to their findings, the pleiotropic statin effect can influence the AAA sac after EVAR and the TIIEL evolution in the follow-up. At 6 months and 36 months, statin patients had higher shrinkage rate and diameter reduction compared with no-statin patients (*P* < .03, *P* < .001). Previous studies have also demonstrated, with histological analysis of the aortic wall in patients on statin therapy, that these drugs are able to reduce the inflammation response and the inflammatory cells and markers and to decrease the protease activity.^27^ Statin therapy is already well established as beneficial for patients with cardiovascular disease; however, these findings further support initiating statins in EVAR patients who were not previously using them, with the aim of reducing EVAR-related complications and the occurrence of TIIEL.

#### Salicylate therapy

According to current guidelines, all patients affected by AAA start antiplatelet therapy right after diagnosis, as AAA is considered pathophysiologically equivalent to coronary artery disease and therefore requires secondary prevention of cardiovascular events.^28^ The study conducted by Álvarez *et al.* demonstrated that salicylate therapy acts as a more effective protector from sac growth compared to other antiplatelet agents. The latter study included patients submitted to EVAR who developed TIIEL during follow-up and found that, 4 years after EVAR, 72% of the nonsalicylate group of patients had an aneurysmal sac growth of 5 mm or more vs only 38% of the patients treated with aspirin or triflusal (*P* < .003). The impact of this therapy on sac growth may be attributed to the anticoagulant properties of salicylates and/or their effects on thrombus composition within the sac; however, the exact protective physiological mechanism is yet to be defined.

#### Smoker status and other factors

The correlation between smoking habit and AAA occurrence and its worsening is proven by numerous studies, stating that smoking has fundamental consequences on sac enlargement and increased rupture risk, caused by not yet defined effects. Koole *et al.* investigated this connection in a 2012 study analyzing nonsmoking (as never smoked or haven’t smoked in at least 10 years), former-smoking patients (patients nonsmoking at the time of the study but smoked in the last 10 years) and actively smoking patients who underwent EVAR treatment. This study highlighted that active smokers have a substantially lower risk of developing TIIEL because of decreased sac perfusion from the mesenteric IMA or lumbar arteries, possibly caused by atherosclerosis impairing the blood flow in these collateral branches. However, sac enlargement or aneurysm neck growth did not differ among these study groups. Fujimura *et al.* conducted a similar study analyzing the influence of smoking habit in TIIEL occurrence in an exclusively East Asian population. This study hypothesized that the lower coagulability observed in East Asian patients would lead to a higher incidence of TIIEL and that the risk factors for TIIEL might differ from those observed in previous Caucasian databases. Non-smoker status was found as an independent risk factor for TIIEL even in this case because of smoke’s atherosclerotic effect on the lumbar artery and IMA and the prothrombotic state caused by smoking lower the incidence of TIIEL.

Sex may represent an additional factor influencing TIIEL development. In the meta-analysis by Guo *et al.*, smoking was inversely associated with TIIEL occurrence (OR 0.71, 95% CI 0.55–0.92), whereas male sex was not significantly associated with TIIEL (OR 0.83, 95% CI 0.67–1.02). However, available studies have not adequately investigated the interaction between sex and smoking status, and whether the inverse association between smoking and TIIEL differs between men and women remains unclear. Further studies incorporating sex-stratified analyses are therefore warranted to clarify this relationship.

Nevertheless, even if smoking habit seems to be associated with a decreased risk of TIIEL development, it is already established as a risk factor for AAA progression, as it increases the vascular wall stress and sac growth.^29^^,^^30^ Therefore, patients with AAA should always be encouraged to stop smoking to slow down disease progression and reduce the risk of associated cardiovascular conditions.

With the same mechanism of smoking, PAD and chronic obstructive pulmonary disease (COPD) were accounted by previous studies as associated with a lower risk of TIIEL.^31^ Both conditions are more prone to the atherosclerosis of collateral vessels responsible for sac expansion and can hypothetically decrease the risk of TIIEL. However, it is important to emphasize that a statistical association alone does not imply a causal relationship. To regard a factor as genuinely “protective” against the occurrence of TIIEL, a plausible physiological rationale and robust supporting evidence are required. Therefore, caution is needed in the interpretation of these results.

## Discussion

Although EVAR is considered a safer option than open surgery for AAA treatment in terms of early postoperative outcomes, it is not free of complications that may necessitate reintervention ^41^. The most frequent of these are endoleaks, particularly TIIEL, which can lead to aneurysmal sac enlargement and, ultimately, EVAR failure.^5^ Although patients treated with EVAR undergo strict surveillance to detect subsequent complications, the early identification of factors associated with TIIEL could substantially reduce their occurrence.^30^ Since the clinical value of EVAR procedures was established, numerous studies over the past three decades have investigated anatomical features and preoperative characteristics that may contribute to EVAR failure. However, recent data on clinical factors potentially associated with TIIEL remain limited.

The findings of the present review highlight that several clinical characteristics may influence the development of TIIEL after EVAR, with anticoagulation emerging as the most consistently reported risk factor, associated with impaired sac thrombosis and persistent sac perfusion. Patients requiring long-term anticoagulation represent a key high-risk subgroup for this complication and should be monitored accordingly. Advanced age also appeared to correlate with increased TIIEL occurrence and, importantly, with a greater likelihood of progression to clinically relevant sac enlargement requiring reintervention.

The present findings should be interpreted in the context of previously published systematic evidence. Guo *et al.* identified advanced age as a significant risk factor for TIIEL, while smoking was inversely associated with its occurrence, findings consistent with those of the present review.^22^ In contrast, anticoagulant therapy was not significantly associated with TIIEL in their analysis. However, incorporating more recent evidence, the present review identified anticoagulation as the most consistently reported clinical risk factor for TIIEL, in accordance with the findings of the systematic review by Kontopodis *et al.*^32^ This discrepancy highlights the evolving evidence regarding clinical predictors of TIIEL and supports the need for updated assessment of patient-related risk and protective factors.

Additional biological contributors, including elevated inflammatory activity detected by FDG-PET/CT and genetic variations affecting extracellular matrix remodeling (such as TIMP-1 rs4898), point to a possible role of inflammation in modulating sac healing. Conversely, several factors such as statin therapy, salicylate use, COPD, PAD, and smoking were reported as potentially “protective”. However, the last three associations are likely influenced by atherosclerotic burden and altered collateral flow rather than true physiological protection.

Future research should prioritize prospective studies aimed at validating these associations, clarifying underlying mechanisms and integrating clinical, biological and imaging markers into robust predictive models. Such efforts are essential to identify meaningful patient subgroups at high risk for TIIEL and to guide personalized follow-up strategies capable of preventing sac enlargement and EVAR failure.

## Conclusion

The identification of clinical risk and protective factors of TIIEL occurrence is extremely relevant in early detection and management of patients possibly at risk of EVAR failure. The present systematic review identified anticoagulant therapy and advanced age as the most consistently reported clinical risk factors for TIIEL after EVAR, while statin and salicylate therapy were associated with a potentially protective effect. Smoking, PAD, and COPD were also inversely associated with TIIEL occurrence, although these findings should be interpreted with caution and should not be considered evidence of a causal protective effect. Considering that TIIEL is the most frequent complication following EVAR, additional research is needed to identify patient subgroups at increased risk, allowing for more tailored treatment and surveillance protocols.

## Data Availability

Data derived from a source in the public domain.

## Abbreviations

AAA, Abdominal Aortic Aneurysm; ABI, Ankle-Brachial Index; COPD, Chronic Obstructive Pulmonary Disease; DOAC, Direct Oral Anticoagulant; EVAR, Endovascular Aneurysm Repair; FDG, Fluorodeoxyglucose; IMA, Inferior Mesenteric Artery; INR, International Normalized Ratio; MeSH, Medical Subject Headings; MMP, Matrix Metalloproteinase; NLR, Neutrophil-to-Lymphocyte Ratio; PAD, Peripheral Artery Disease; PET/CT, Positron Emission Tomography/Computed Tomography; PLR, Platelet-to-Lymphocyte Ratio; PRISMA, Preferred Reporting Items for Systematic Reviews and Meta-Analyses; TIIEL, Type II Endoleak; TIMP-1, Tissue Inhibitor of Metalloproteinases-1
